# Serotyping and antibiotic susceptibility patterns of *Vibrio* and *Shigella* isolates from diarrheal patients visiting a Tropical and Infectious Diseases Hospital in central Nepal

**DOI:** 10.1186/s13104-017-2967-0

**Published:** 2017-11-28

**Authors:** Sujan Maharjan, Binod Rayamajhee, Anima Shreshtha, Jyoti Acharya

**Affiliations:** 1 St. Xavier’s College (Tribhuvan University), Maitighar, Kathmandu, Nepal; 2 Kathmandu Research Institute for Biological Sciences (KRIBS), 19533, Lalitpur, Nepal; 3 Department of Microbiology, National College (Tribhuvan University), Khusibu, Kathmandu, Nepal; 4 Tri-Chandra Multiple College (Tribhuvan University), Ghantaghar, Kathmandu, Nepal; 5 National Public Health Laboratory, Government of Nepal, Ministry of Health Services, Teku, Kathmandu, Nepal

**Keywords:** Diarrheal disease, Antimicrobial resistance, Serotype

## Abstract

**Background:**

Diarrheal diseases are the major infectious disease in developing countries like Nepal. Lack of proper sanitation and antimicrobial resistance gained by microbes have challenged to address diarrheal diseases in resource-limited countries. Early diagnosis of disease and proper antibiotic treatment can significantly reduce the disease burden. This study was designed to determine the recent antimicrobial susceptibility pattern of *Vibrio cholerae* and *Shigella* spp. to assure the proper antibiotic treatment. Stool specimens were processed following microbiological protocol and identified by biochemical and serological tests recommended by the Clinical Laboratory Standard Institute.

**Results:**

Out of total 640 analyzed stool samples, 50 were culture positive, among them 29 were *Shigella* spp. (64.4%) and 21 were *V. cholerae* (46.6%). All *V. cholerae* strains belonged to the serogroup O1 and serovar Ogawa. Among the *Shigella* spp., *Shigella flexneri* 17 (59%) topped the list of serotype followed by *Shigella sonnei* 8 (28%), *Shigella dysenteriae* 3 (10%) and *Shigella boydii* 1 (3%) respectively. All the *V. cholerae* isolates (100%) were sensitive to cefotaxime while 71% were sensitive to tetracycline but 100 and 90.4% were resistance to co-trimoxazole and nalidixic acid respectively. *Shigella* isolates were mostly susceptible to cefotaxime (97%) while ciprofloxacin (48%) and ofloxacin (55%) were less effective drugs.

**Conclusions:**

These results on the prevalence of enteropathogens and their antibiotic resistance pattern may help to guide accurate choice of therapy in clinical setting. Hence, development of evidence based National Guidelines for the treatment of diarrhea is needed.

**Electronic supplementary material:**

The online version of this article (10.1186/s13104-017-2967-0) contains supplementary material, which is available to authorized users.

## Background

Enteric diseases are being cardinal cause of morbidity and mortality in the resource limited countries like Nepal which is considered as the second leading cause of death in children under 5 years and around 525,000 children are losing their life each year due to enteric diseases [1]. Outbreak of diarrheal diseases including cholera epidemics is being a cardinal health problem in Nepal since long time ago. Burden of diarrheal diseases is reported throughout the year but outbreaks are seen during and immediately after the rainy season of every year [2]. The incidence of diarrheal diseases in Nepal increases during the summer season with the high preponderance in July and August months; beginning with the monsoon rain in May. As being a communicable disease which transmits via contaminated water and food, diarrheal diseases become more active during the monsoon season [3, 4].

*Shigella* is one of the leading causes of bacterial diarrhea in Nepal and contains four species *flexneri*, *sonnei*, *dysenteriae*, and *boydii*. In Mid and Far Western parts of the country *Shigella flexneri* has been the predominant isolate with 43.07% [5]. The prevalence of *Shigella* species causing an acute diarrhea in children under 5 years of age in Kathmandu valley is 4.6% [6]. Similarly, cholera is a secretory diarrheal disease that causes excessive fluid secretion which can result from bacterial enterotoxin (cholera toxin) of *Vibrio cholerae* strains. More than 150 serogroups of *V. cholerae* have been confirmed but the toxigenic serogroup O1 of *V. cholerae* is the cardinal causative serogroup of several outbreaks since long time ago [7]. All the isolates of *V. cholerae* were serogroup O1 of the classical biotype where subtype was Ogawa [8]. During cholera outbreak in Far-Western part of Nepal in 2013, all the isolates were *V. cholerae* having prevalence of 47.06% [9].

Antimicrobial resistance (AMR) is one of the greatest challenge to the public health locally and globally and has gained particular concern in the developing world. Herein Nepal, inappropriate use of antibiotics is very common which creates an environment where drug-resistant microbes flourish very rapidly. Additionally, the resistance of enteric bacteria to antibiotics has been increasing very sharply both in developed and developing communities [10]. The rate of AMR in our health setting is alarming and the preparation for its future calamities is almost zero. AMR has seen even to new antimicrobial drugs and is very commonly observed in bacteria like *V. cholerae*, *Shigella*, *Salmonella*, and *Staphylococcus aureus* [11]. In context of Nepal, underuse, misuse and overuse of antibiotics without health care professional advice, availability of antibiotics even in local groceries, use of antibiotics in farm and field, sharing of antibiotics with family and friends are very common which accelerate the development of antimicrobial resistance [12]. Thus, the main objective of this study was to identify *Shigella* spp., *V. cholerae* and monitor the changing antimicrobial susceptibility pattern in Nepal.

## Methods

This study was carried out in Sukraraj Tropical and Infectious Disease Hospital, from June 2014 to December 2014. A total of 650 stool samples were processed from the patients who had been consulted with the clinician were included as the study subject. The stool samples were transferred to microbiology laboratory and processed as soon as possible according to the standard laboratory methods. In this study, patients have remained in hospital for longer than 3 days and/or in prior antibiotic use were kept in exclusion criteria.

For the isolation of *V. cholerae*, a loopful of fresh watery stool was inoculated on alkaline peptone water (APW) (pH 8.6) in a volume of 1:10. In case of swab it was dipped into the alkaline peptone water tube directly and incubated aerobically for 6 h at 37 °C. After incubation, precaution was taken to avoid shaking of the enrichment culture which may disturb the growth at the surface of the broth while solid and semi-solid stool samples were directly inoculated on xylose lysine deoxycholate agar (XLD) and MacConkey agar (MA) then incubated at 37 °C for 24 h. Similarly, after 6 h the APW inoculated samples were streaked on thiosulfate citrate bile salts sucrose (TCBS) and MA then incubated at 37 °C for 24 h. After proper incubation period, the TCBS plates were observed for the presence of yellow button shaped sucrose fermenting colonies while XLD plates were observed for the pink red colonies of 1–2 mm diameter with no black center and late lactose fermenting colonies on MA. Then, the pathogenic organism was further sub-cultured on nutrient agar for isolation of pure colonies at 37 °C for 24 h and appropriate biochemical tests (catalase, oxidase, urease, sulfide indole motility, triple sugar iron, citrate) were performed [13]. The *V. cholerae* was confirmed as sucrose fermenting yellow colonies on TCBS, late lactose fermenting colonies on MA, oxidase positive, catalase positive, urease negative, glucose and sucrose fermentative, maltose non-fermentative, citrate positive, indole positive, methyl red negative, Voges–Proskauer negative, hydrogen sulfide (H_2_S) negative, and in triple sugar iron agar tube acid/acid with no gas production (identification of *V. cholerae* CDC, 2012). The pink colonies from XLD and late lactose fermenting colonies from MA were tested for urease production, citrate utilization, indole production, motility, acid and gas production with sugar utilization. *Shigella* spp. were confirmed based on biochemical results along with oxidase and urease negative tests (identification of *Shigella* spp. Public Health England, 2015). While serological identification was done for the *Shigella* spp. for the identification of specific species using the polyvalent antisera A–D (*Shigella* Antisera) and *V. cholerae* (polyvalent O1, Inaba, Ogawa and Hikojima type monovalent antisera) by slide agglutination method with the use of specific antisera manufactured by Denka Seiken Co. Ltd., Japan. Slide agglutination was performed on a glass slide by emulsifying the growth in a small drop of physiological saline and mixed thoroughly by tilting back and forth for 30 s. Small drop of antiserum was added to the respective suspension. The suspension and antiserum were mixed well and tilted back and forth to observe for visible agglutination. One drop of physiological saline and the growth emulsion was used as a negative control and to observe for auto-agglutination (Denka Seiken Co. Ltd., Japan). Details of the serotyping by slide agglutination method is included in Additional file 1: Appendix S1.

Antibiotic susceptibility test of the isolated and identified strains was performed by modified Kirby Bauer disc diffusion method; a standard dilution of the test isolate was prepared by matching it with 0.5 McFarland turbidity standards and was uniformly swabbed over the Mueller Hinton agar (MHA) medium. Then antibiotic discs (Hi-media, Mumbai, India): ampicillin (10 µg), cotrimoxazole (1.25 µg/23.5 µg), nalidixic acid (30 µg), cefotaxime (30 µg), chloramphenicol (30 µg), ciprofloxacin (5 µg), ofloxacin (5 µg), and tetracycline (30 µg) were placed on the medium and incubated at 37 °C for 24 h according to the isolated organism. After incubation period the zone of inhibition were measured and results were interpreted as per the guidelines given by the Clinical Laboratory Standard Institute (CLSI) [14].

## Results

**Fig. 1:**
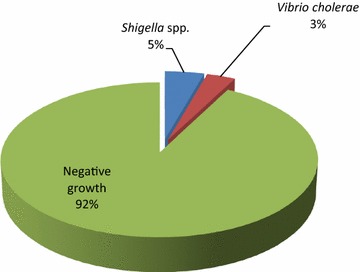
Distribution of the positive cases in total samples. From total of 650 stool samples analyzed, 50 samples were found to be positive for enteric bacterial pathogens of which 29 (5%) were *Shigella* spp. and 21 (3%) were *Vibrio cholerae* where total positive case was 7.7%

From total of 650 stool samples processed, 50 samples were found to be positive for enteric bacterial pathogens of which 21 (3%) were *V. cholerae* and 29 (5%) were *Shigella* spp. where total positive case was 7.7% (Fig. 1).

Serotyping results showed that all the strains of *V. cholerae* agglutinate polyvalent O1 antisera hence all *V. cholerae* belonged to serogroup O1 and serotype Ogawa while non of them were neither Inaba nor Hikojima serotype. Out of 29 *Shigella* species isolated, *S. flexneri* was the highest with 17 (59%), followed by *Shigella sonnei* 8 (28%), *Shigella dysenteriae* 3 (10%) where *Shigella boydii* was the least isolated with 1 (3%) (Table 1).

**Table 1:** Result of serotyping of isolated *Shigella* species

*Shigella* serogroup	*Shigella* species	Number of cases	Percentage %
Polyvalent A	*Shigella dysenteriae*	3	10
Polyvalent B	*Shigella flexneri*	17	59
Polyvalent C	*Shigella boydii*	1	3
Polyvalent D	*Shigella sonnei*	8	28

**Fig. 2:**
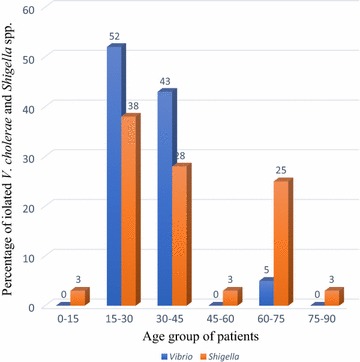
Age wise distribution of gastroenteritis. The patients of age group 15–30 years were mostly affected by both cholera (52%) and shigellosis (38%)

Among 650 samples, the highest positive cases of gastroenteritis was seen in ward patients (in-patients) of 82%, followed by out-patients with 18% and no positive cases was seen in patients living with human immuno deficiency virus (PLHIV) (Table 2). There was no significant difference in occurrence between gastroenteritis cases and type of the patients (P > 0.05). The patients of age group 15–30 years were mostly affected by both cholera (52%) and shigellosis (38%) (Fig. 2). Among 50 positive cases, 28 (56%) were isolated from male while 22 (44%) were isolated from female patients (Table 3) where there was no significant differences in between growth of the pathogen and gender of patients (P > 0.05).

**Table 2:** Patients wise distribution of gastroenteritis

Patient type	Result		Total	P value
	Negative cases	Positive cases		
Out patients	170	9 (18%)	179	0.12
In patients	414	41 (82%)	455	
PLHIV	16	0 (0%)	16	
Total	600	50	650

**Table 3:** Gender wise distribution of gastroenteritis

Gender	Result		Total	P value
	Negative	Positive		
Male	274	28	302	0.15
Female	326	22	348	
Total	600	50	650	

**Fig. 3:**
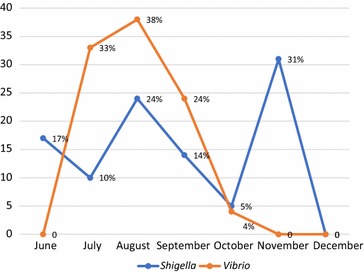
Month wise distribution of gastroenteritis from June 2014 to Dec 2014. The distribution of bacterial pathogens of gastroenteritis showed increment in rainy season and sharp decrement in winter. The peak value of cholera positive cases were seen in the month of August with a steady curve graph. The highest number of shigellosis was seen in November with a zig zag pattern of graph plot

The distribution of bacterial pathogens of gastroenteritis showed increment in rainy season and sharp decrement in winter. The peak value of cholera positive cases were seen in the month of August with a steady curve graph. The highest number of shigellosis was seen in November with a zig zag pattern of graph plot (Fig. 3).

All the isolated *V. cholerae* were found to be sensitive to cefotaxime (CTX), while 71% were sensitive and 29% were intermediately sensitive to tetracycline (TET). All the strains were completely resistant to cotrimoxazole (COT) and 90% were resistant to nalidixic acid (NA) (Table 4). Almost all the *Shigella* isolates in this study were susceptible to cefotaxime (97%) except one isolate which was found to be resistant to this drug. The isolated *Shigella* spp. were mostly found to be resistant to nalidixic acid (NA) (76%), ampicillin (AMP) (58%), and cotrimoxazole (55%). Ciprofloxacin (CIP) and ofloxacin (OF) which are used as a drug of choice were found to be 48 and 55% sensitive for *Shigella* spp. whereas resistance was 52 and 41% for ciprofloxacin and ofloxacin respectively (Table 5).

**Table 4:** Antibiotic susceptibility pattern of *Vibrio cholerae*

Total positive cases	Antibiotics	Sensitive (%)	Intermediate (%)	Resistant (%)
	Ampicillin	–	33	67
	Cotrimoxazole	–	–	100
	Nalidixic acid	–	10	90
21	Cefotaxime	100	–	–
	Chloramphenicol	57	33	10
	Ciprofloxacin	81	19	–
	Tetracycline	71	29	–

**Table 5:** Antibiotic susceptibility pattern of *Shigella* spp.

Total positive cases	Antibiotics	Sensitive (%)	Intermediate (%)	Resistant (%)
	Ampicillin	21	21	58
	Cotrimoxazole	41	4	55
	Nalidixic acid	17	7	76
29	Cefotaxime	97	–	3
	Chloramphenicol	86	7	7
	Ciprofloxacin	48	–	52
	Ofloxacin	55	4	41

### Statistical analysis

Chi square test was used to evaluate apparent differences for significance at 95% confidence level. Association of bacterial isolates with different variables were tested. Results were considered significant if P value was less than 0.05 and insignificant if more than 0.05. Statistical Package for the Social Sciences (SPSS version 21.0) was used for the Chi square test.

## Discussion

This study helps to understand the prevalence of enteric pathogens which is related to the public health concern in developing countries like Nepal and the need of antibiotic susceptibility assay for proper treatment and to control the emergence of resistant organisms.

In this study, 650 stool samples were processed for the isolation of *V. cholerae* and *Shigella* spp. and their antimicrobial sensitivity test was performed. Out of these, 50 positive cases were found, of which 21 cases were *V. cholerae* and 29 cases were *Shigella* spp., resulting in the total positive case of 7.7%. The causes of diarrhea in patients without positive bacterial culture might be parasitic infections, bacteria other than *V. cholerae* and *Shigella* spp., or foodborne infection and intoxication. The result had a similar prevalence with the study carried out by Ansari et al. [6]. Moreover, Kansakar et al. [15] have observed 15% culture positivity for bacterial enteropathogens from stool samples. Since, the prevalence of enteric disease varies from place to place while proper sanitation and hygiene of the people affect the occurrence of the disease. Bacterial pathogens could have been missed in the single stool sample processed so, second sample for stool culture should be made available to rule out the negative culture [16]. Also antibiotics are often consumed in Nepal without consulting the physicians, so administration of antibiotics prior to collection of stool samples may be one of the causes of 7.7% growth positivity.

The prevalence rate of cholera was 3.2% and is comparable to findings of Kansakar et al. [15] and Shah et al. [17]. The prevalence rate of shigellosis was 4.5% which is comparable to Shah et al. [17], Kansakar et al. [15] and Ansari et al. [6]. The highest number of positive cases were seen in the inpatients (hospitalized) or ward patients of 41 (82%) and 9 (18%) were from outpatients while no pathogenic bacteria were isolated from PLHIV patients. There was no statistical association between the type of patients and the gastroenteritis cases (P > 0.05). Out of 50 positive cases, 56% were male and 44% were female. Thus, there is sufficient evidence to say that there was no association between sex and the incidence of the disease (P > 0.05). In this study male patients were slightly more infected than female and age wise distribution of gastroenteritis cases showed that the cases were high in the age group 15–30 which is then followed by 30–45 and 60–75. There was no significant difference in occurrence of gastroenteritis cases and the age group (P > 0.05). The cause of high number of male patient with age group 15–30 may be due to the fact that the people of this age group are of working age group and the unhygienic food and water may have caused the infection of enteric diseases. Also highest number of cholera cases was observed during the rainy season and peak value was seen in August whereas shigellosis was seen almost all over the study period with highest number of cases in November (31%). In Nepal, rainy season starts from early June and lasts till September. The arrival of monsoon brings up the number of acute diarrheal diseases and cholera is a major diarrheal disease among them. During the study period all the isolated strains of *Vibrio* (21) were *V. cholerae* serogroup O1 serotype Ogawa. In recent years, Ogawa is predominant serotype of *V. cholerae* O1 during epidemics season. *V. cholerae* O1 serotype Ogawa and of biotype El Tor was the only serotype isolated during the outbreak of diarrhea in Kavre, Nepal in 2004 [18] and same serotype was isolated during 2008–2009 in a laboratory based surveillance study conducted by Karki et al. [19].

Among 29 *Shigella* spp. isolated*, S. flexneri* was the most predominant species with 17 (59%), then next were *S. sonnei* 8 (28%), *S. dysenteriae* 3 (10%) where *S. boydii* was the least isolated with 1 (3%). From this study, it is observed that *S. flexneri* was the common species responsible for causing dysentery followed by *S. sonnei, S. dysenteriae* and *S. boydii*. The similar finding was obtained in the study conducted by Kansakar et al. [15] in which *S. flexneri* was the most prevalent species (43.2%) but the second highest species was found *S. dysentrieae* (41.5%), followed by *S. sonnei* (7.6%) and *S. boydii* (7.6%).

In this study, antibiotic susceptibility test (AST) for the isolated and identified species was performed by modified Kirby Bauer disc diffusion method for seven different antibiotic discs. The AST result shows that all the isolates of *V. cholerae* were found to be sensitive to cefotaxime (CTX), while 71% were sensitive and 29% were intermediately sensitive to tetracycline (TET). The isolated *V. cholerae* were found to be 100, 90, and 67% resistant to cotrimoxazole, nalidixic acid, and ampicillin in decreasing order. Similar findings were reported during *V. cholerae* O1 Ogawa strains isolated in 2008 in Kathmandu by Karki et al. [19]. This study showed that tetracycline may still be used as the drug of choice in case of severe cholera cases as none of the isolated showed resistant to this drug. But still the antibiotic susceptibility test should be performed prior to drug prescription which reduce the antibiotic resistance and disease burden.

Almost all the *Shigella* isolates in this study were susceptible to cefotaxime (97%) except one isolate which was found to be resistant to this drug. Ciprofloxacin and ofloxacin which are used as a drug of choice were found to be 48 and 55% sensitive for this organism respectively whereas resistance was 52 and 41% for ciprofloxacin and ofloxacin respectively. Ciprofloxacin is considered as the drug of choice for all kind of patients with bloody diarrhea but resistance has seen in many parts of the world. Cefotaxime and other third generation cephalosporins are currently in use for the treatment of multi-drug resistant (MDR) species of *Shigella* while azithromycin is used as an alternative drug for the treatment of young adults. The prescription of these alternative drugs like ceftriaxone and azithromycin is restricted because of high rate of resistance in resource limited countries like Nepal. Generally alternative drugs are used when the isolated strains of *Shigella* are resistant to ciprofloxacin [20].

## Conclusions

This study shows that the isolated bacterial enteric pathogens were *V. cholerae* O1 serovar Ogawa and *Shigella* spp. which includes *S. flexneri, S. sonnie, S. dysenteriae,* and *S. boydii*. Tetracycline and ciprofloxacin showed an adequate effectiveness against the isolated strains of *V. cholerae*. In this study the isolated *Shigella* spp. were mostly resistant to nalidixic acid followed by ampicillin, cotrimoxazole, ciprofloxacin, and ofloxacin while cefotaxime was found to be most effective drug. So, regular laboratory monitoring of antimicrobial susceptibility pattern is becoming cardinal in order to choose the accurate drug and in designing evidence-based National Guidelines for the treatment of diarrheal patients in developing regions.

## Additional file

**Additional file 1: Appendix S1.** Determining serotypes of *Vibrio* and *Shigella* species.

## Abbreviations

AST: antibiotic susceptibility test
WHO: World Health Organization
NCID: National Center for Infectious Diseases
CDC: Centers for Disease Control and Prevention
USAID: United States Agency for International Development
CLSI: Clinical Laboratory Standard Institute
PLHIV: patients living with human immunodeficiency virus
APW: alkaline peptone water
GE: gastroenteritis
MHA: Mueller–Hinton agar
µg: micro gram
