# Supplementary material for: Serotyping and antibiotic susceptibility patterns of Vibrio and Shigella isolates from diarrheal patients visiting a Tropical and Infectious Diseases Hospital in central Nepal
Source: BMC Res Notes. 2017 Nov 28;10:626. doi: 10.1186/s13104-017-2967-0 (PMC5704626; doi:10.1186/s13104-017-2967-0)
Supplement: Supplementary file 1 — Additional file 1: Appendix S1. Determining serotypes of Vibrio and Shigella species. [file 13104_2017_2967_MOESM1_ESM.docx]

**Appendix S1**

***Vibrio* antisera “DENKA SEIKEN”**

Products

Set: 4 vials (polyvalent O1, Inaba, Ogawa and Hikojima type monovalent antisera).

Denka Sieken Co. Ltd., 3-4-2 Nihonbashikayaba-Cho, Chuo-Ku, Tokyo, Japan.

**Serotyping method of *Vibrio* *cholerae***

1. One drop (10 µl) of polyvalent O1 antisera was dropped on the clean glass slide. Then *Vibrio cholerae* colony was picked by inoculating loop and mixed with antisera. Then it was observed for agglutination. If agglutination appears, then further testing with monovalent antisera was done.
2. Serotyping with monovalent antisera Inaba, Ogawa and Hikojima were done to screen out the serotype of the O1 *Vibrio cholerae.* The agglutination with respective antiserum identify that the strain related to that serotype.
3. For non-agglutinating*Vibrio cholerae* with polyvalent O1 antiserum, further serotyping with O139 antiserum was done.
4. The suspension without adding antisera was kept as control to detect auto agglutination.

***Shigella* antisera “DENKA SEIKEN”**

Isolated *Shigella* colonies were mixed with polyvalent antiserum of *S. sonnei*, *S. dysenteriae*, *S. flexneri*, *S. boydii* and results were recorded accordingly.

**Product: Polyvalent sera**

| **S.N.** | **Polyvalent serum** | **Sub-groups** |
| --- | --- | --- |
| **Subgroup A (*S. dysenteriae*)** | Polyvalent A | Mixture of type 1-7 in subgroup A |
|  | Polyvalent A1 | Mixture of type 8-12 in subgroup A |
| **Subgroup B (*S. flexneri*)** | Polyvalent B | Mixture of each type 1-7 and group in subgroup B |
| **Subgroup C (*S. boydi*)** | Polyvalent C | Mixture of type 1-7 in subgroup c |
|  | Polyvalent C1 | Mixture of type 8-11 in subgroup C |
|  | Polyvalent C2 | Mixture of type 12-15 in subgroup C |
|  | Polyvalent C3 | Mixture of type 16-18 in subgroup C |
| **Subgroup D (*S. sonnei*)** | Polyvalent D | Mixture of phase I and phase II |
